# Carboxymethyl Chitosan Modified Oxymatrine Liposomes for the Alleviation of Emphysema in Mice via Pulmonary Administration

**DOI:** 10.3390/molecules27113610

**Published:** 2022-06-04

**Authors:** Jianqing Peng, Zimin Cai, Qin Wang, Jia Zhou, Jinzhuan Xu, Di Pan, Tingting Chen, Guangqiong Zhang, Ling Tao, Yi Chen, Xiangchun Shen

**Affiliations:** 1 The High Efficacy Application of Natural Medicinal Resources Engineering Center of Guizhou Province, School of Pharmaceutical Sciences, Guizhou Medical University, University Town, Guian New District, Guiyang 550025, China; pengjianqing90@126.com (J.P.); caizimin97@126.com (Z.C.); wangqin_yx@163.com (Q.W.); zhoujia_gy@163.com (J.Z.); xujinzhuan_yx@163.com (J.X.); pandipharm@gmc.edu.cn (D.P.); zgq_2021@126.com (G.Z.); taoling202001@126.com (L.T.); 2 The Key Laboratory of Optimal Utilization of Natural Medicine Resources, School of Pharmaceutical Sciences, Guizhou Medical University, University Town, Guian New District, Guiyang 550025, China; 3 Guiyang Maternal and Child Health Care Hospital, Guiyang 550003, China; chen_tingting66@126.com

**Keywords:** oxymatrine, emphysema, carboxymethyl chitosan, pulmonary delivery, liposomes

## Abstract

Pulmonary emphysema is a fatal lung disease caused by the progressive thinning, enlargement and destruction of alveoli that is closely related to inflammation and oxidative stress. Oxymatrine (OMT), as a bioactive constituent of traditional Chinese herbal *Sophora flavescens*, has great potential to alleviate pulmonary emphysema via its anti-inflammatory and antioxidative activities. Pulmonary administration is the most preferable way for the treatment of lung diseases. To improve the in vivo stability and pulmonary retention of OMT, OMT-loaded liposome with carboxymethyl chitosan (CMCS) modification was developed. The CMCS was modified on the surface of OMT liposomes via electrostatic attraction and covalent conjugation to obtain Lipo/OMT@CMCS and CMCS-Lipo/OMT, respectively. A porcine pancreatic elastase (PPE)-induced emphysema mice model was established to evaluate the alleviation effects of OMT on alveolar expansion and destruction. CMCS-modified liposomal OMT exhibited superior ameliorative effects on emphysema regardless of the preparation methods, and higher sedimentation and longer retention in the lung were observed in the CMCS-Lipo group. The mechanisms of OMT on emphysema were related to the downregulation of inflammatory cytokines and the rebalancing of antioxidant/oxidation via the Nrf2/HO-1 and NF-κB/IκB-α signaling pathways, leading to reduced cell apoptosis. Moreover, the OMT liposomal preparations further enhanced its anti-inflammatory and antioxidative effects. In conclusion, pulmonary administration of OMT is a potential strategy for the treatment of emphysema and the therapeutic effects can be further improved by CMCS-modified liposomes.

## 1. Introduction

Pulmonary emphysema is caused by damage to the air sacs in the lungs and is characterized by the gradual enlargement and destruction of alveoli in the lung, which is one of the lung conditions that causes breathing difficulties [1]. Although the pathogenesis of emphysema is not fully understood, accumulating evidence suggests that inflammation and oxidative stress play important roles in the damage of lung parenchyma, leading to emphysema [2,3,4]. When lung tissue is exposed to exogenous poisons, inflammatory cells such as neutrophils and macrophages are recruited across vascular endothelium and accumulate in the pulmonary alveoli, which further promotes the secretion of inflammatory cytokines [5]. Furthermore, the breakage of oxidation/antioxidation balance results in oxidative stress and inflammatory responses as well [6]. Moreover, inflammation and oxidative stress result in cell death and damage to the lung extracellular matrix, leading to the destruction of the alveolar wall and lung parenchyma [7]. As clinical treatments for emphysema by corticosteroids suffer from steroid refractoriness, oral candidiasis and cataracts [8,9], the discovery of novel therapeutic regimens to alleviate emphysema is needed.

Oxymatrine (OMT) is a quinazoline alkaloid extracted from Sophora flavescens. Previous studies have revealed that OMT exerts anti-inflammatory, antioxidative stress and anti-apoptotic effects on various diseases, such as liver failure [10], diabetic nephropathy [11] and myocarditis [12]. The anti-inflammatory effect of OMT is mainly achieved through the nuclear factor-kappa B (NF-κB) signal pathway, which can inhibit the phosphorylation-NF-κB-p65 to ameliorate inflammatory responses [13]. Moreover, OMT has been reported to reduce oxidative damage by upregulating the nuclear factor erythroid 2-related factor (Nrf2), which is an important antioxidative transcription factor [14]. Moreover, OMT could protect the damaged tissues by reducing apoptosis by increasing the ratio of Bcl-2/Bax. Because of the activation of NF-κB, an increased level of Bax and downregulation of Nrf2 and Bcl-2 have been reported in the lung tissue of emphysema patients [15,16], thus, OMT has the great potential to alleviate emphysema via modulating these signaling pathways.

For the treatment of respiratory diseases, pulmonary administration is preferred due to its advantages in escaping from first-pass metabolism and directly targeting delivery to lung tissue, leading to high bioavailability, rapid absorption, and fewer adverse effects [17,18]. Nevertheless, pulmonary administrated medicines have to overcome various physiologic barriers, including pulmonary clearance, enzymatic degradation, and a short half-life [19]. Nano-sized drug delivery systems have been employed to address these challenges in pulmonary administration [20]. Liposome, with great biocompatibility and safety, has been widely used in the pulmonary delivery of both hydrophilic and hydrophobic drugs, which could protect the drug from enzymatic degradation and prolong pulmonary retention [21]. The OMT-encapsulated liposome was first reported in the treatment of hepatitis [22]. It was verified with improved in vivo stability, prolonged circulation, and accumulation of OMT at the targeted organs [23,24,25], which is a promising nanoformulation of OMT for pulmonary administration.

Surface modification is a promising strategy for preventing liposomes from fast elimination by fast blood flow and immune recognition, resulting in an improved half-life in vivo [26]. Carboxymethyl chitosan (CMCS), a widely used biological adhesive material, is a good candidate for pulmonary drug delivery systems due to its high biocompatibility and biodegradability [27,28]. It is expected to form a hydration shell on the surface of liposomes to protect them from degradation during nebulization and in the bronchus. Both physical absorption and the covalent conjugation method were adopted in the surface coating of liposomes. Hence, CMCS, with a negative charge, can bind with positively charged liposomes via electrostatic attraction or covalently conjugate with an amino end on the surface of liposomes via an amide bond. Although CMCS-coated nanoparticles, which are prepared by both physical and chemical methods, have been reported for pulmonary administration [29,30,31], it is unclear which surface modification method is more suitable for OMT-encapsulated liposomes to achieve the best therapeutic effects on emphysema.

**Scheme 1:**
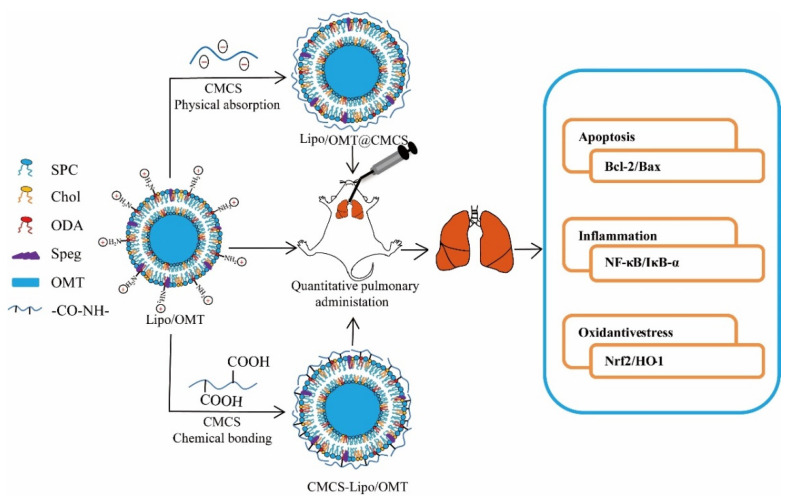
Schematic illustration for the preparation of OMT liposomal preparations and therapeutic effects on emphysema in mice.

In this study, we developed two CMCS-modified OMT liposomes via physical adsorption and covalent conjugation, respectively, for pulmonary administration. The ameliorative effects of OMT liposomal preparations on an emphysema mice model were primarily evaluated, and the mechanisms were explored in terms of anti-inflammation and antioxidation (Scheme 1). This study provides new insight into the development of a liposomal formulation of hydrophilic drugs for pulmonary administration.

## 2. Materials and Methods

### 2.1. Materials and Reagents

Oxymatrine (OMT), polyoxyethylene stearate (SPEG), rhodamine B (RB), 1-(3-Dimethylaminopropyl)-3-ethylcarbodiimide hydrochloride (EDCI) and carboxymethyl chitosan (CMCS, N-linked, carboxylation degree 80%) were offered by Macklin Biochemical Co., Ltd. (Shanghai, China), respectively. Octadecylamine (ODA) and N-Hydroxysuccinimide (NHS) were supplied by Adamas-beta (Shanghai, China). Soybean phospholipid (SPC) was supplied by A.V.T Pharmaceutical Co., Ltd. (Shanghai, China). Cholesterol (Chol) was purchased from VetecTM reagent grade (Shanghai, China). Porcine pancreatic elastase (PPE) was purchased from Macklin Biochemical Co., Ltd. (Shanghai, China).

### 2.2. Animals

Six-week-old C57BL/6 mice of either sex (18–22 g) were supplied by the Experimental Animal Center of Guizhou Medical University. All animal care and experiments have been approved by the Animal Welfare and Ethics Committee of Guizhou Medical University (No: 1804102). Mice were raised at room temperature (23 ± 2 °C) and constant humidity (45 ± 10%) in an SPF condition. One week before starting the experiments, animals were placed for adaptation.

### 2.3. Preparation of CMCS-Coated OMT Liposomal Preparations

The OMT liposomal preparations were prepared using the pH-gradient method. In brief, SPC, Chol, ODA and SPEG were dissolved in dichloromethane at a mass of 10:2:1:1 and evaporated from lipid film under reduced pressure for 30 min using a rotary evaporator (RE52CS, Shanghai Yarong biochemical instrument, Shanghai, China). The lipid film was resuspended with 0.1 M critic acid buffer (pH 4.0) and then incubated in a water bath for 30 min at 60 °C. Next, the film was hydrated using a water bath sonicator (200 W) for 3 min. The obtained Lipo were extruded through a 0.45 μm filter. After the formation of the empty Lipo, the external pH was adjusted to pH 7.0 with a 2 M NaOH solution. Then, OMT was added to the Lipo solution. The Lipo/OMT were then incubated for 30 min at 40 °C with occasional shaking. For the preparation of Lipo/OMT@CMCS, CMCS was coated on a liposomal surface through electrostatic adsorption. CMCS solution, dissolved in phosphate-buffered saline (PBS) at a concentration of 20 mg/mL, was added dropwise to the freshly prepared Lipo/OMT solution at a volume ratio of 1:60 and stirred at 500 rpm for 30 min. Furthermore, CMCS-Lipo/OMT was obtained via an amide reaction. The carboxyl groups in CMCS were activated by EDCI and NHS, and then the activated carboxyl groups were bonded to primary amines in ODA [32]. In brief, 3.3 mg of EDCI and 2.0 mg of NHS were added to the CMCS solution for activation for 1 h, and then mixed with Lipo/OMT at the same ratio and stirred for 12 h. The free OMT was removed via dialysis PBS to obtain Lipo/OMT and CMCS-coated OMT liposomal preparations.

The RB-encapsulated liposomes were prepared via a film dispersion method. Briefly, 4 mL of RB solution at 0.5 mg/mL was incubated with the thin lipid film in a water bath at 60 °C for 30 min. The Lipo/RB was obtained after passing through a 0.45 μm filter. After the modification of CMCS via the same methods described above, Lipo/RB@CMCS and CMCS-Lipo/RB were obtained.

### 2.4. Characterization of OMT Liposomal Preparations

The morphology of Lipo/OMT, Lipo/OMT@CMCS and CMCS-Lipo/OMT were observed by transmission electron microscope (TEM, Tecnai 12, Philips, Eindhoven, Holland). The particle diameter, polydispersity (PDI), and zeta potential of Lipo/OMT, Lipo/OMT@CMCS and CMCS-Lipo/OMT were measured by a NanoBrook 90Plus PALS (Brookhaven, NY, USA). The concentration of the OMT in liposomal preparations was assayed by high-performance liquid chromatography (HPLC), which used an Agilent 1260 HPLC system with a reversed-phase column Hanbon C98200305228 (4.6 × 250 mm, 5 μm, Huai’an, China) at 30 °C. The mobile phase consisted of acetonitrile and 0.2% triethylamine (8:92, *v/v*) at a flow rate of 1 mL/min and was detected at a wavelength of 210 nm. Equations (1) and (2) were used to calculate the drug loading efficiency (LE) and loading capacity (LC). The in vitro release profile was measured using the dialysis method. A dialysis bag (MWCO, 14 KDa) holding 2 mL of OMT-encapsulated liposomal preparations was immersed in 15 mL of PBS in a capped bottle and incubated at 37 °C with stirring at a speed of 50 rpm. At a predetermined time point, 500 μL of the medium was withdrawn and replenished with fresh PBS at the same volume. The amount of OMT released into the medium was determined using the HPLC method described above. The release medium satisfied the sink conditions, and the experiments were performed in triplicate. To determine the stability of the formulations, Lipo/OMT, Lipo/OMT@CMCS and CMCS-Lipo/OMT were kept in an incubator at 4 °C for one week. The particle size, PDI and zeta potential were measured over the storage periods.

(1) $$\mathrm{LC}(\%)=\frac{W_{\mathrm{drug}\;\mathrm{encapsulated}}}{W_{\mathrm{total}\;\mathrm{Liposome}}+W_{\mathrm{drug}\;\mathrm{encapsulated}}}\times 100$$

(2) $$\mathrm{LE}(\%)=\frac{W_{\mathrm{drug}\;\mathrm{loaded}}}{W_{\mathrm{drug}\;\mathrm{added}}}\times 100$$

### 2.5. In Vivo Biodistribution of RB-Encapsulated Liposomal Preparations

Organ distributions were obtained after pulmonary administration of free RB, Lipo/RB, Lipo/RB@CMCS and CMCS-Lipo/RB. A handheld aerosolizer (MicroSprayer^®^ Aerosolizers, Yuyan instruments Co., Ltd., Shanghai, China) was used for the intratracheal spraying of free RB and RB-labeled liposomes as previously described [33]. Briefly, mice were anesthetized by CO_2_ inhalation and put in a supine position. Once the sprayer needle was inserted into the trachea to reach the carina, it was retraced 1–2 mm to avoid the splash of the suspension. Then, 50 μL of suspension was administered in one push. Above all, the standard curves of plasma and various organs (heart, liver, spleen, lung, kidney) were established. Then, the mice were grouped randomly and quantitively pulmonary administered with free RB and RB-labeled liposomes at an RB dose of 750 μg/kg. Different organs and blood samples were collected at the fixed intervals of 0.5, 1, 2, 4 and 8 h. The lung tissue was homogenized in PBS and the plasma was separated by centrifugation at 10,000× *g* for 10 min. Ethanol was added to the homogenates and plasma under constant stirring at 500 rpm for 30 min, respectively. Then, the mixture was centrifuged at 3000× *g* for 15 min and the supernatant was determined via a fluorescence spectrophotometer (Varian Cary Eclipse, Palo Alto, CA, USA).

### 2.6. Treatments on PPE-Induced Emphysema in Mice

We performed a mice model of emphysema induced by PPE, as previously described [34]. Mice received PPE (10 U in 100 μL saline) by intratracheal spraying once weekly for 4 weeks to establish an emphysema model. The intratracheal spraying was conducted with MicroSprayer^®^ Aerosolizers, as mentioned above. Mice were randomly assigned to six groups: the control group, challenged with 100 μL of saline solution by intratracheal spraying once weekly for 4 weeks; the model group, which received PPE by the same route, in the corresponding times and volume. For the intervention with medication during this period: the OMT group received 12.5 mg/kg OMT by intratracheal spraying every other day; the Lipo/OMT group received a corresponding dose of Lipo/OMT by the same route and time; the Lipo/OMT@CMCS group received a corresponding dose of Lipo/OMT@CMCS by the same route and time; the CMCS-Lipo/OMT group received a corresponding dose of CMCS-Lipo/OMT by the same route and time. After treatment, the mice were euthanized under deep anesthesia and the blood samples were collected through the abdominal aorta. Subsequently, the left main bronchus of mice was ligated, and then the trachea was incised and inserted into a cannula; bronchoalveolar lavage fluid (BALF) was collected on the right lung of mice in each group by washing three times with 0.8 mL of ice-cold PBS. Then, the right lung was carefully excised and stored at −20 °C for biochemical analyses and Western blotting. The left lung was removed and inflated in 4% paraformaldehyde (Biosharp, Beijing Labgic Technology Co., Ltd., Beijing, China) for histological analysis.

### 2.7. Analysis of Bronchoalveolar Lavage Fluid (BALF)

The BALF from each mouse was centrifuged at 4000× *g* for 10 min at 4 °C, and the sedimented cells were resuspended in 0.5 mL of PBS and dropped onto the slide. After drying, Wright Giemsa dye was added for staining. Two hundred leukocytes were counted under the optical microscope, and the proportion of macrophages, neutrophils and lymphocytes was recognized via morphological characteristics and calculated. Briefly, the cytoplasm of the neutrophils was in the form of pink, rod-shaped nuclei with variable leaves and was mainly trilobal with uniform and fine granules in the cytoplasm. In comparison to the neutrophils, the macrophages were larger and possessed a more rounded soma and cytoplasm, with fine and loose chromatin, and were relatively pale in coloration. The nuclei of lymphocytes are large, dark purple, and densely stained with minimal cytoplasm [34,35,36].

### 2.8. Histopathology and Cell Apoptosis Evaluation in Lung Tissue

The fixed left lung tissues of mice were dehydrated in a series of graded ethanol and embedded in paraffin. The slides of 5 μm thickness were stained with hematoxylin and eosin (H&E) staining for morphometric and histopathological evaluation. The mean linear intercept (MLI) was assessed by qualifying the airspace enlargement and destruction. The MLI was calculated by counting the number of alveolar walls crossed by the reticulum in ten randomly selected fields of tissue sections per lung sample as previously described [35]. Furthermore, the terminal deoxynucleotidyl transferase-mediated dUTP nick end labeling (TUNEL) was used to identify apoptotic cells in mice lung tissue by a commercially available kit (Servicebio, G1501, Wuhan, China). The fluorescent staining was examined using the Panoramic MIDI (3DHISTECH Ltd., Budapest, Hungary). The results were expressed as the ratio of the TUNEL-positive cell number to the total number of cells counted.

### 2.9. Determination of Inflammatory Cytokines and Oxidative Stress Indicators

The plasma was separated from the blood samples by centrifugation at 10,000× *g* for 10 min. Tumor necrosis factor-α (TNF-α), interleukin 1β (IL-1β) and interleukin 6 (IL-6) were quantified in plasma samples using a specific enzyme-linked immunosorbent assay (ELISA) with a mouse anti-mouse monoclonal antibody. The procedure complied with the manufacturer’s instructions (Fanyin, Shanghai, China).

The lung tissue was homogenized in PBS and centrifuged at 3500× *g* for 10 min, the supernatants were collected and dissolved in extraction buffer for the analysis of malondialdehyde (MDA), superoxide dismutase (SOD), and catalase (CAT) content. The total protein in the sample was determined using the BCA Protein Assay Kit (Solarbio, Beijing, China). To examine the level of lipid peroxidation in the lung tissue, the MDA concentration was assessed by the lipid peroxidation MDA Assay Kit (Beyotime, S0131, Shanghai, China). For antioxidative enzyme activities in the lung tissue, SOD and CAT levels were detected with a total SOD Assay Kit with WST-8 (Beyotime, S0101) and a CAT assay kit (Jiancheng Bioengineering Institute, A007-1-1, Nanjing, China).

### 2.10. Western Blot Analysis

Lung tissues were lysed in radioimmunoprecipitation assay buffer (Solarbio, R0010, Beijing, China) containing 1% protease inhibitor phenylmethylsulfonyl fluoride (Solarbio, P0100, Beijing, China). The lung samples were homogenized and centrifuged at 10,000× *g* for 10 min. The supernatant was collected, and the protein concentrations were evaluated using the BCA Protein Assay Kit (Solarbio, Beijing, China) according to the manufacturer’s instructions. The protein sample (45 μg) was resolved on sodium dodecyl sulfate-polyacrylamide gel electrophoresis and electrotransferred to polyvinylidene difluoride membranes (Millipore, Bedford, MA, USA). Subsequently, primary antibodies against Nrf2, HO-1, B-Cell Leukemia/Lymphoma 2 (Bcl2), BCL2 Associated X (Bax), IκB alpha (IκB-α) and beta-actin (β-actin) were purchased from ProteinTech (ProteinTech group, catalog number:66504-1, 10701-1-AP, 12789-1-AP, 50599-2, 10268-1-AP, 66009-1-lg). Primary antibodies against phosphor-NF-κB p65 antibody were purchased from Cell Signaling Technology (Cell Signaling Technology, catalog number:3033S). After incubating with the corresponding secondary antibody, the proteins were performed using an enhanced chemiluminescence kit (7 Sea Biotech, Shanghai, China) and Syngene Gel Imaging system (Bio-Rad, Hercules, CA, USA). The levels of proteins in the membranes were quantified by Image Lab software (Bio-Rad, Hercules, CA, USA).

### 2.11. Statistical Analysis

Data are presented as mean ± SD. Comparisons between the experimental groups were assessed by an unpaired, two-tailed Student’s *t*-test. Multiple groups were made using one-way ANOVA followed by a post hoc Tukey test among the model and treatment groups. The significance was defined as *p*-values of less than 0.05.

## 3. Results and Discussion

### 3.1. Preparation and Characterization of CMCS-Modified Liposomal OMT

In the preparation of OMT liposomes (Lipo/OMT), the weight ratio of SPC: Chol and SPC: OMT was optimized in terms of LE and LC of OMT (Figure S1a,b). Chol affects the mechanical properties of the liposome membranes by increasing the mechanical strength, elasticity and density of the lipid bilayer through ordering and condensation effects [37]. An increased ratio of Chol restricts the transmembrane transport of small molecules, which are closely related to the LE of drug. At the SPC: the Chol weight ratio was 5:1, and the highest LE of OMT was received. The amount of hydrophilic drug in liposomes partly depends on the volume of an internal aqueous phase, thereby, the size of the liposomes positively correlates with the LE of the drugs [38]. The increased SPC/OMT weight ratio showed an improved LE of OMT and an optimum LE was obtained at an SPC/OMT weight ratio of 5:1. The LC and LE of the OMT liposomes were greatly affected by the incubation temperature and time as well (Figure S1c,d). The maximum LC and LE were received at 40 °C and 30 min incubation.

**Figure 1:**
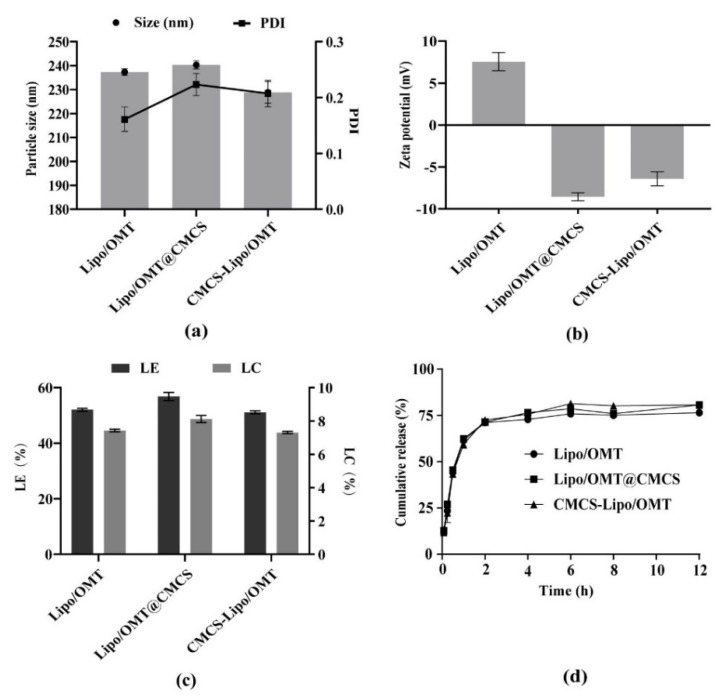
Characterization of OMT liposomal preparations. (**a**) Evolution of particle size, PDI and (**b**) zeta potential of OMT liposomal preparations. (**c**) Drug loading efficiency and loading capacity of OMT in different liposomal preparations. (**d**) In vitro release behavior of OMT from different liposomal preparations in pH 7.4 PBS. All data are expressed as mean ± SD (*n* = 3).

Upon OMT encapsulation and CMCS modification, the particle size, PDI and zeta potential of the OMT liposomal preparations were evaluated (Figure 1a,b). The particle size of Lipo/OMT and Lipo/OMT@CMCS are 237.34 ± 1.46 nm and 240.31 ± 1.71 nm, respectively. While a slightly reduced particle size of 228.82 ± 4.53 nm was detected for CMCS-Lipo/OMT. The PDI values of all the OMT liposomal preparations are less than 0.3, indicating uniformity of liposomes. The zeta potential of Lipo/OMT was 7.65 ± 1.09 mV. Owing to the negative charge of CMCS, the zeta potential of the CMCS-modified liposomes shifted to a negative charge. The LE and LC of the OMT liposomal preparations were approximately 53% and 8%, respectively (Figure 1c). The morphology of the OMT liposomal preparations was observed by TEM images (Figure S2).

To evaluate the stability of the OMT liposomal preparations, particle size, PDI and the zeta potential of the OMT liposomal preparations stored at 4 °C were detected for 7 days (Figure S3). A reasonable variation in the particle size, PDI or zeta potential in all the groups indicated good stability of the liposomal preparations with no aggregation formed. Moreover, in vitro drug release studies were performed to study the release behavior of encapsulated OMT and around 80% of OMT was released after 4 h in all the OMT liposomal preparations (Figure 1d). CMCS modification maintained the stability of liposomes in vitro while failing to retard the release of OMT. It is hypothesized that a CMCS coating forms a shell on the periphery of liposomes which can prevent destruction by enzyme and endogenous material, while the intermolecular channels of CMCS might not be narrow enough to restrict the leakage of OMT.

### 3.2. In Vivo Distribution of RB-Encapsulated Liposomal Preparations

**Figure 2:**
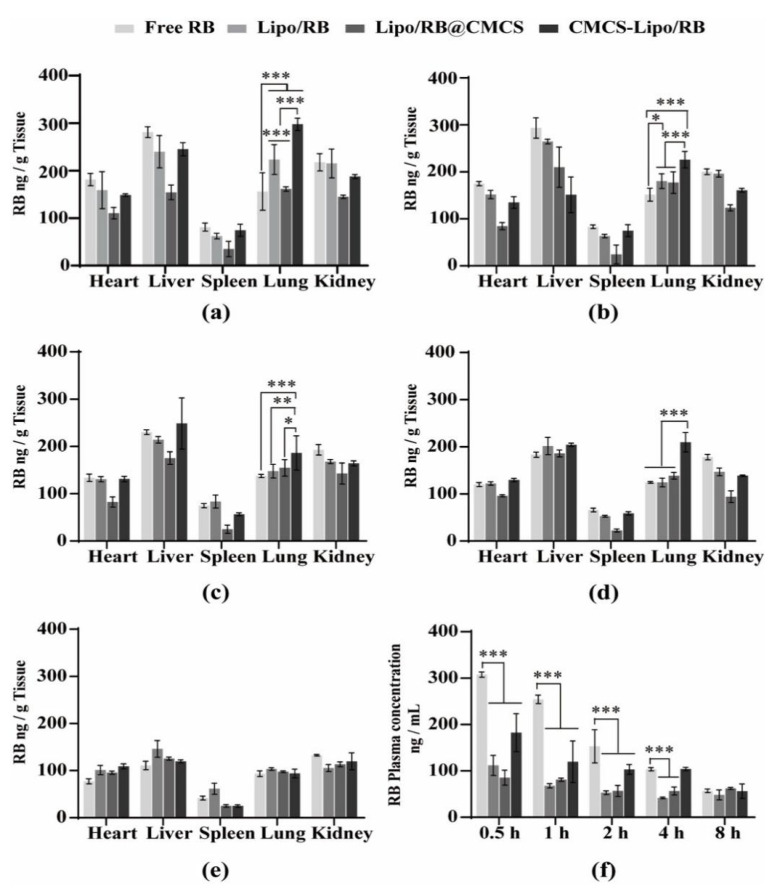
Biodistribution of RB in mice at (**a**) 0.5 h, (**b**) 1 h, (**c**) 2 h, (**d**) 4 h and (**e**) 8 h after quantitative pulmonary administration of RB preparations and (**f**) the plasma concentration of RB. All the data were expressed as mean ± SD (*n* = 6). * *p* < 0.05, ** *p* < 0.01, *** *p* < 0.001.

The pulmonary sedimentation of preparations is closely related to the diameter of nebulized droplets, which mainly depends on the device for pulmonary administration [39]. RB-encapsulated liposomal preparations were prepared to investigate the in vivo biodistribution of liposomes and CMCS-modified liposomes after pulmonary administration (Figure 2). RB was used as a fluorescent surrogate for estimating the biodistribution of OMT due to its similar hydrophilicity and loading within the liposome liposomal preparation that promoted the sedimentation of RB, and it was further enhanced by covalently conjugated CMCS. Whereas, the CMCS coating, via physical adsorption, failed to improve the sedimentation of RB, which might be contributed to the destruction of Lipo/RB@CMCS during transportation across the bronchus. Moreover, CMCS-Lipo/RB exhibited obviously longer retention in the lung than in the other groups until 8 h. Correspondingly, the liposomal preparations showed a much lower plasma concentration of RB, indicating less systematic escape. It is suggested, however, that the increased sedimentation and retention of encapsulated RB in the lung from CMCS-Lipo/RB might be attributed to the successful protection of liposomes to avoid degradation during the nebulization and in the bronchus, which provided the foundation for improving the alleviative effects of OMT on emphysema.

### 3.3. Alleviative Effects of OMT Liposomal Preparations on PPE-Induced Emphysema Mice

**Figure 3:**
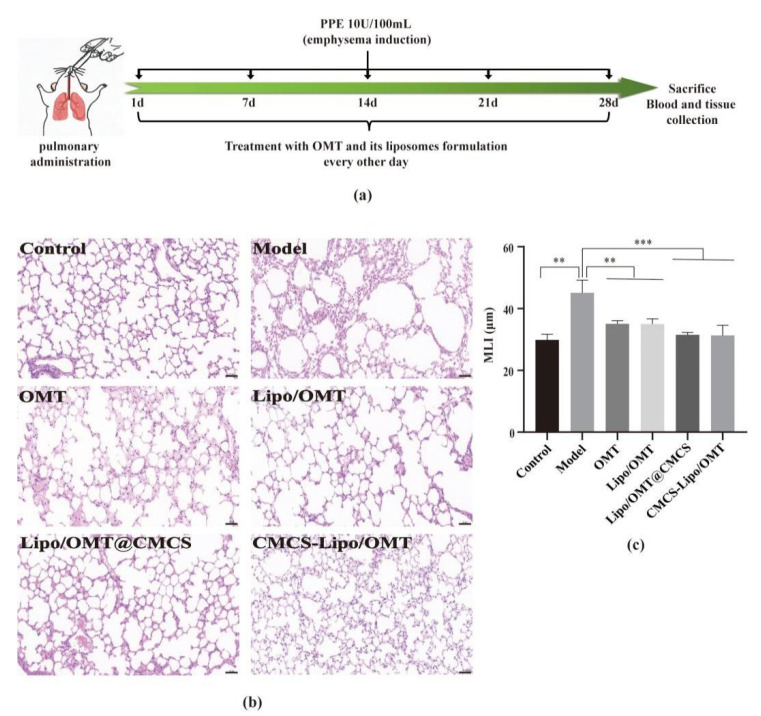
Effect of OMT and its liposomal preparations on the structure of lung tissue in PPE-induced emphysema mice. (**a**) A scheme of experimental protocol for the establishment of an emphysema model and therapeutic regimen. (**b**) Representative histology images of the lung sections from each group, stained with H&E (magnification, ×200; Scale bar = 50 μm). (**c**) Quantitation of mean linear intercept (MLI) of lung tissue. All data are expressed as mean ± SD (*n* = 3). ** *p* < 0.01, *** *p* < 0.001.

We investigated the effects of OMT and its liposomal preparations on PPE-induced emphysema in mice via quantitative pulmonary administration. A scheme of the experimental protocol is shown in Figure 3a. The emphysema mouse model induced by PPE has been used to study pharmacological preventive and curative treatments for 40 years, which shows similar pathological findings of emphysema in patients, including ruptured alveolar walls, dilated airspaces and inflammatory cell infiltration [40]. In the present study, the histological changes in lung tissues were assessed by H&E-stained sections (Figure 3b). Normal lung tissue presented fine interlacing alveoli, while PPE-treated mice exhibited thickened alveoli septa, dilated alveoli, and an increased accumulation of inflammatory cells. As one of the indicators for clinical assessment of emphysema, MLI, which represents the mean free distance between gas exchange surfaces in the acinar airway complex, was measured (Figure 3c). The MLI of the model group significantly increased than that of the control group, indicating the successful establishment of PPE-induced emphysema mice. Free OMT treatments via pulmonary administration prevented the rupture of alveolar walls and formation of bullae compared with the model group, resulting in lower MLI. Similar amelioration effects were observed in the Lipo/OMT group. It was further improved in the Lipo/OMT@CMCS and CMCS-Lipo/OMT groups, which showed comparable MLI to the control group. It is suggested that the destructed lung structure induced by PPE might be attenuated by OMT preparations via pulmonary administration, especially for CMCS-modified liposomes.

### 3.4. Suppression of Cell Apoptosis by OMT Liposomal Preparations

**Figure 4:**
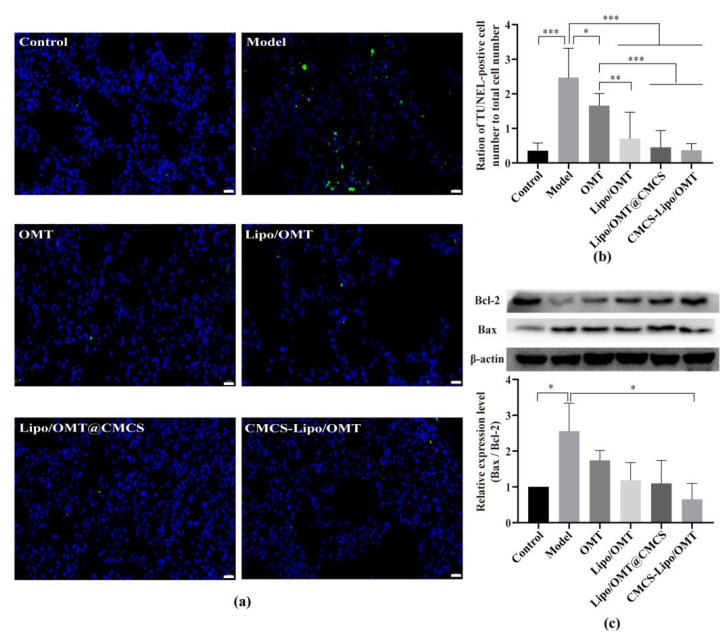
Regulation of OMT and its liposomal preparations on PPE-activated alveolar cell death in emphysema mice lungs. (**a**) Representative lung section images of each group, analyzed by TUNEL assay (scale bar = 20 µm), in which the apoptotic alveolar cell apoptosis (green) and nuclei (blue) were labeled. (**b**) Changes in the ratio of TUNEL-positive cell number to total cell number in the lung sections of each group of mice. (**c**) Western blotting analysis and quantitative analysis of the relative expression of Bax/Bcl-2 in lung tissues. All data are expressed as mean ± SD (*n* = 3). * *p* < 0.05, ** *p* < 0.01, *** *p* < 0.001.

Apoptosis is a key process associated with the development of emphysema [41]. We examined whether apoptosis was increased in PPE-induced emphysema and the protective effect of OMT preparations by performing TUNEL staining of lungs. TUNEL-positive cells increased in the lungs of model mice compared with the control group, and the alveolar septal cell occupied the predominant cell type. Treatment with OMT preparations significantly prevented the increases of TUNEL-positive cells in the lung of PPE-treated mice. OMT liposomal preparations could further reduce the proportion of TUNEL-positive cells compared with OMT, especially in the Lipo/OMT@CMCS and CMCS-Lipo/OMT groups (Figure 4a,b). To investigate the molecular basis of reducing cell apoptosis in the lungs of emphysema mice by OMT preparations, the expression of Bcl-2 and Bax was detected (Figure 4c). Consistent with the results of apoptosis, the ratio of Bax to Bcl-2 was remarkably elevated in the emphysema model group compared with the control group and it was reduced by the OMT preparations. Intriguingly, a significant difference was only detected between CMCS-Lipo/OMT and the model group. It is indicated that OMT preparations exert an anti-apoptosis activity by regulating the expression level of the Bax and Bcl-2, which might prevent the destruction of pulmonary parenchymal.

### 3.5. Inhibition of the Inflammation and Oxidative Stress in Emphysema Mice

**Figure 5:**
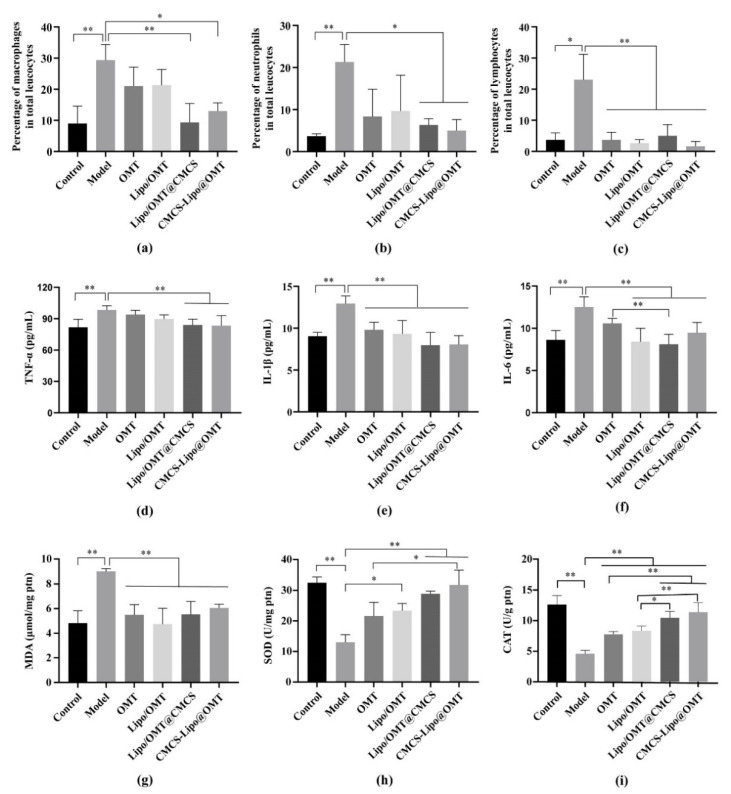
Effect of OMT and its liposomal preparations on the inflammation and oxidation stress in PPE-induced emphysema mice. The percentage of (**a**) macrophages, (**b**) neutrophils and (**c**) lymphocytes in total leucocytes of BALF. The level of (**d**) TNF-α, (**e**) IL-1β and (**f**) IL-6. The levels of (**g**) MDA, (**h**) SOD and (**i**) CAT. (**a**–**c**) Data represent the means ± SD (*n* = 3). (**d**–**i**) Data are expressed as mean ± SD (*n* = 5). * *p* < 0.05, ** *p* < 0.01.

Accumulating evidence shows that overwhelmed inflammatory responses and excessive oxidative stress play essential roles in the pathogenesis of emphysema [42,43]. Above all, we evaluated the proportion of immune cells in the BALF by using the Wright–Giemsa staining methods to determine the inflammation level in the lung of PPE-treated mice (Figure 5a–c). An increased proportion of macrophages, neutrophils and lymphocytes were detected in the BALF of model mice compared with that in the control group, which were reduced by the OMT preparations. CMCS-modified OMT liposomes further downregulated the proportion of macrophages, neutrophils and lymphocytes in the BALF of mice. Given that lymphocytes gather in the lung might induce chemokines release, leading to apoptosis of epithelial and alveolar cells [44,45], the reduced recruitment of lymphocytes was closely related to the sharply decreased cell apoptosis. As inflammatory cytokines are strongly related to the development of emphysema [46], we analyzed the level of long-term inflammatory mediators in the plasma by ELISA assay (Figure 5d,f). Similarly, the level of TNF-α, IL-6 and IL-1β were significantly higher in PPE-treated mice than in the control group, and the OMT preparations suppressed the expression of inflammatory cytokines. However, no obvious difference was observed among the OMT liposomal preparations. Our results suggested that pulmonary administration of the OMT preparations effectively alleviated the progression of inflammation.

The accumulation of macrophages and neutrophils in the lung of model mice not only boosts inflammatory cytokines and cell release but also enhances the production of reactive oxygen species (ROS). The PPE-treatment leads to an excessive generation of MDA and reduced activity of antioxidant enzymes SOD and CAT in the lung of mice (Figure 5g–i). After being treated with OMT preparations, it showed significantly decreased MDA in all the groups at a similar level and remarkable improvement in the activity of SOD and CAT, especially in the CMCS-Lipo/OMT group, which was comparable to that of the control group. Collectively, OMT exhibited anti-inflammatory and antioxidant effects on the PPE-induced emphysema in mice via pulmonary administration, and liposomal preparations further enhanced the effects of OMT, especially after CMCS modification. It is suggested that the improved anti-inflammatory and antioxidant effects of OMT are attributed to the prolonged retention in the lung with the help of CMC-modified liposomes.

### 3.6. Mechanisms of Suppression on Inflammation and Oxidative Stress

To further investigate the protective mechanisms of OMT and its liposomal preparations on PPE-induced emphysema, the expression of NF-κB and Nrf2 was detected. Researchers found that the expression of NF-κB p65 is significantly increased in emphysema patients and animal models [47,48]. As an important transcription factor that is closely related to inflammation, NF-κB plays a key role in regulating the immune response and cytokine activity [49]. The activation of NF-κB p65 is restricted by the interactions with IκBα. In response to external stimuli, IκBα is ubiquitinated and degraded by proteasomes, leading to the phosphorylation of NF-κB and translocation to the nucleus, which results in inflammatory responses [50]. In terms of oxidative stress, Nrf2, as a nuclear transcription activator, regulated the induction of defensive genes encoding the detoxifying enzymes and antioxidant proteins [51]. HO-1 is a downstream protein of Nrf2, which showed protective effects against oxidative stress [52]. According to the previous reports, decreased expression of Nrf2 was observed in the lung of emphysema patients, while the therapeutic regimens that maintained or promoted the level of Nrf2 was potential therapy for emphysema [53].

**Figure 6:**
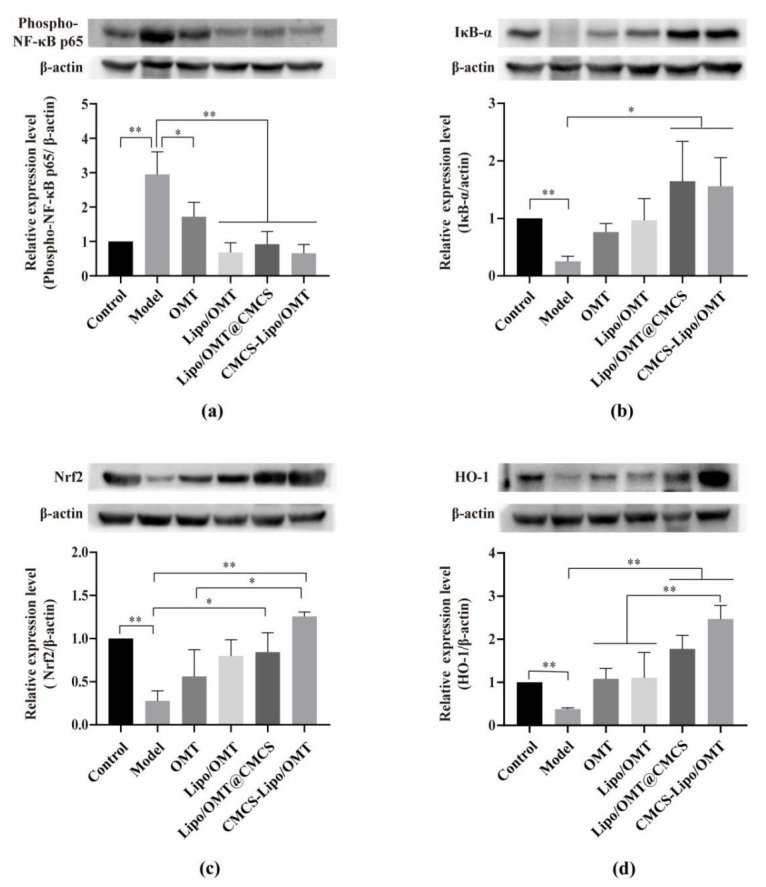
The protein expression of (**a**) phosphor-NF-κB p65, (**b**) IκB-α, (**c**) Nrf2 and (**d**) HO-1 in the lung tissues of emphysema mice after treatment by OMT preparations. All data are expressed as mean ± SD (*n* = 3). * *p* < 0.05, ** *p* < 0.01.

In our study, Nrf2, HO-1 and IκBα were markedly reduced and phosphorylated NF-κB p65 was significantly elevated in the PPE-stimulated emphysema model in comparison with that of the control group (Figure 6). Pulmonary administration of OMT and Lipo/OMT slightly increased the expression of Nrf2, HO-1 and IκBα, however, there was no significant difference compared with the model group. CMCS-modified liposomes significantly improved the expression of Nrf2, HO-1 and IκBα, especially for the CMCS-Lipo/OMT group, regardless of the similar levels of phosphorylated NF-κB p65 in the OMT liposomal preparations. These results indicated that OMT may play an important role by inhibiting the dissociation of NF-κB P65 and IκBα and exerting antioxidative effects via increasing the expression of Nrf2 and its downstream.

In summary, pulmonary administration of OMT could exert its ameliorative effects in emphysema mice by regulating NF-κB p65 and Nrf2, which suppressed the inflammatory reactions and oxidative stress. Its anti-inflammatory and antioxidant effects were increased after liposome encapsulation and CMCS modification. Moreover, covalent conjugated CMCS on the periphery of liposomes exhibited longer retention in the lung and superior antioxidant effects. It is hypothesized that covalent conjugation of CMCS might provide a more stable OMT liposomal preparation compared with the CMCS modification via electrostatic adsorption. However, no significance was detected between the CMCS-modified OMT liposomes in terms of MLI, apoptosis, and anti-inflammation.

## 4. Conclusions

In the present study, we prepared Lipo/OMT by a pH-gradient method and two CMCS-modified Lipo/OMTs via electrostatic attraction (Lipo/OMT@CMCS) and covalent conjugation (CMCS-Lipo/OMT). It was found that pulmonary administration of OMT could exert its anti-inflammatory and antioxidant effects to alleviate PPE-induced emphysema by regulating the Nrf2/HO-1 and NF-κB p65 signaling pathways. CMCS-modified OMT liposomal preparations enhanced the therapeutic effects of OMT on emphysema. Despite the superior sedimentation and retention of CMCS-Lipo/RB in the lung than Lipo/RB@CMCS, no significant difference was detected between Lipo/OMT@CMCS and CMCS-Lipo/OMT in the treatment of emphysema. Hence, CMCS-modified liposome might be a promising drug delivery system for pulmonary administration of OMT to treat emphysema.

## Data Availability

Not applicable.

## Supplementary Materials

The following supporting information can be downloaded at: https://www.mdpi.com/article/10.3390/molecules27113610/s1, Figure S1: Optimization of Lipo/OMT on the (a) weight ratio of SPC to Chol, (b) weight ratio of SPC to OMT, the incubation time (c) and temperature (d) in terms of LE and LC of OMT. Each bar represents the means ± SD (*n* = 3); Figure S2: Representative TEM images of Lipo/OMT (scale bar 100 nm), Lipo/OMT@CMCS and CMCS-Lipo/OMT (scale bar 200 nm). Figure S3: The changes in particle size (a), PDI (b) and zeta potential (c) of OMT liposomal preparations stored at 4 °C for 7 days. Each bar represents the means ± SD (*n* = 3).

## Sample Availability

Samples of the compounds are not available from the authors.
