# Supplementary material for: Carboxymethyl Chitosan Modified Oxymatrine Liposomes for the Alleviation of Emphysema in Mice via Pulmonary Administration
Source: Molecules. 2022 Jun 4;27(11):3610. doi: 10.3390/molecules27113610 (PMC9182538; doi:10.3390/molecules27113610)
Supplement: Supplementary file 1 [file molecules-27-03610-s001.zip › molecules-1749135-supplementary.pdf]

Supporting Information

Carboxymethyl Chitosan Modified Oxymatrine Liposomes for the Alleviation of Emphysema in Mice via Pulmonary Administration

Jianqing Peng [1,2,†], Zimin Cai [1,2,†], Qin Wang [1,2,†], Jia Zhou [1,2], Jinzhuan Xu [1,2], Di Pan [1,2], Tingting Chen [3], Guangqiong Zhang [1,2], Ling Tao [1,2], Yi Chen [1,2,*] and Xiangchun Shen [1,2,*]

[1] The High Efficacy Application of Natural Medicinal Resources Engineering Center of Guizhou Province, School of Pharmaceutical Sciences, Guizhou Medical University, University Town, Guian New District, Guiyang 550025, China; pengjianqing90@126.com (J.P.); caizimin97@126.com (Z.C.); wangqin_yx@163.com (Q.W.); zhoujia_gy@163.com (J.Z.); xujinzhuan_yx@163.com (J.X.); pandipharm@gmc.edu.cn (D.P.); zgq_2021@126.com (G.Z.); taoling202001@126.com (L.T.)

[2] The Key Laboratory of Optimal Utilization of Natural Medicine Resources, School of Pharmaceutical Sciences, Guizhou Medical University, University Town, Guian New District, Guiyang 550025, China

[3] Guiyang Maternal and Child Health Care Hospital, Guiyang 550003, China; chen_tingting66@126.com

* Correspondence: chenyi_19890319@126.com (Y.C.); shenxiangchun@126.com (X.S.); Tel.: 86-0851-88416153 (Y.C.); +86-0851-88174180 (X.S.)

† These authors contributed equally to this work.

Figures

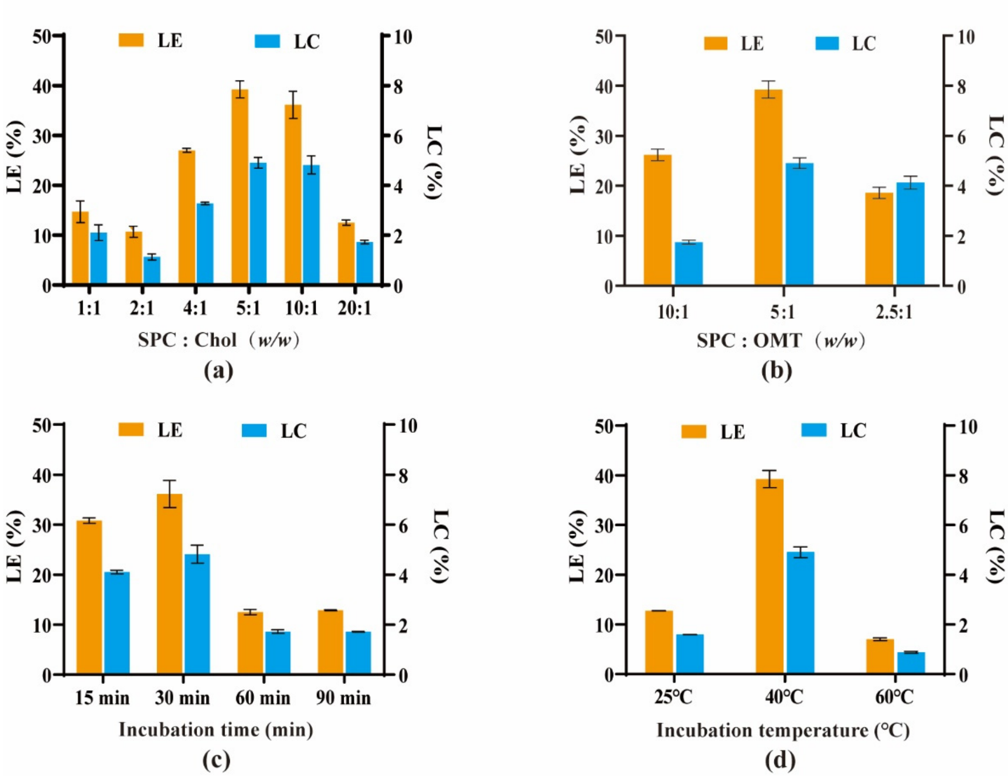

Figure S1 Optimization of Lipo/OMT on the (a) weight ratio of SPC to Chol, (b) weight ratio of SPC to OMT, the incubation time (c) and temperature (d) in terms of LE and LC of OMT. Each bar represents the means ± SD ($n = 3$).

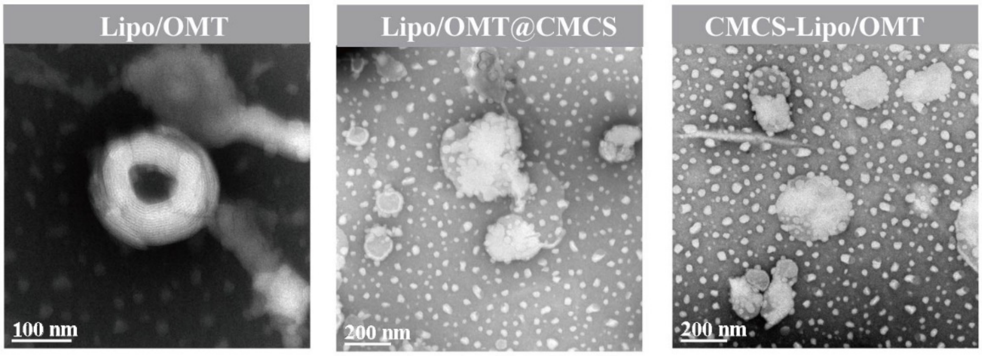

Figure S2 Representative TEM images of Lipo/OMT (scale bar 100 nm), Lipo/OMT@CMCS and CMCS-Lipo/OMT (scale bar 200 nm).

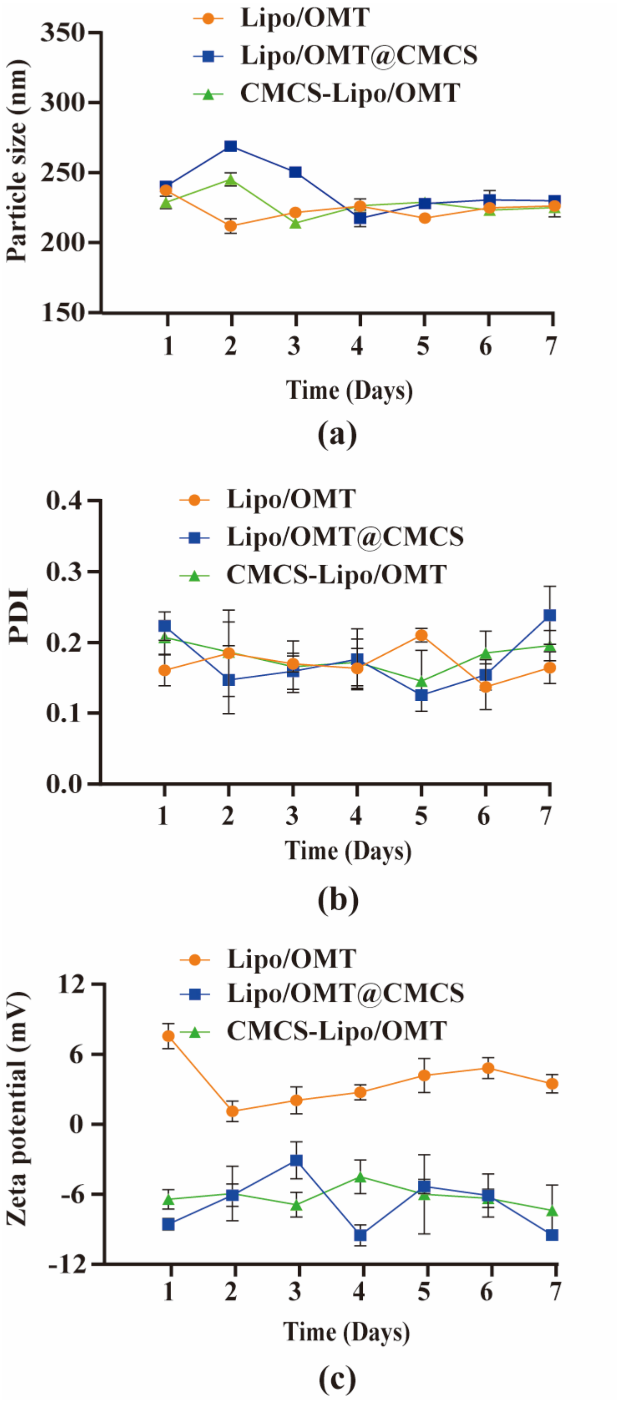

Figure S3 The changes in particle size (a), PDI (b) and zeta potential (c) of OMT liposomal preparations stored at 4°C for 7 days. Each bar represents the means ± SD (n = 3).
